# Orientation-Specificity of Adaptation: Isotropic Adaptation Is Purely Monocular

**DOI:** 10.1371/journal.pone.0047425

**Published:** 2012-11-07

**Authors:** John Cass, Ameika Johnson, Peter J. Bex, David Alais

**Affiliations:** 1 School of Psychology, University of Western Sydney, Penrith, New South Wales, Australia; 2 School of Psychology, University of Sydney, Sydney, Australia; 3 Schepens Eye Research Institute, Harvard Medical School, Cambridge, Massachusetts, United States of America; University of Sussex, United Kingdom

## Abstract

Numerous studies have found that prolonged exposure to grating stimuli reduces sensitivity to subsequently presented gratings, most evidently when the orientations of the adapting and test patterns are similar. The rate of sensitivity loss varies with angular difference indicating both the presence and bandwidths of psychophysical ‘orientation channels’. Here we study the orientation dependency of contrast adaptation measured both monoptically and dichoptically. Earlier psychophysical reports show that orientation bandwidths are broader at lower spatial frequencies, and we confirm this with a simple von Mises model using 0.25 vs. 2 c.p.d. gratings. When a single isotropic (orientation invariant) parameter is added to this model, however, we find no evidence for any difference in bandwidth with spatial frequency. Consistent with cross-orientation masking effects, we find isotropic adaptation to be strongly low spatial frequency-biased. Surprisingly, unlike masking, we find that the effects of interocular adaptation are purely orientation-tuned, with no evidence of isotropic threshold elevation. This dissociation points to isotropic (or ‘cross-orientation’) adaptation being an earlier and more magnocellular-like process than that which supports orientation-tuned adaptation and suggests that isotropic masking and adaptation are likely mediated by separate mechanisms.

## Introduction

Orientation selectivity is a common feature of neural response in early visual cortex and is well-established across a range of mammalian species at both single-cellular and population levels of analysis [1]–[4]. In human observers, orientation-tuning characteristics of neuronal populations have been inferred using psychophysical techniques. A popular psychophysical paradigm used to infer orientation-selectivity is overlay masking, which involves measuring changes in the visibility of an oriented target stimulus when it has an additional (masking) stimulus spatio-temporally superimposed upon it [5]–[9]. Typically, target sensitivity is reduced maximally when the target and mask have the same orientation, but progressively less so as the angular difference between target and mask increases. This rate of sensitivity change with angular difference provides an index of orientation-selectivity (tuning), with steeper dependencies associated with greater selectivity.

Another method for studying orientation tuning is adaptation. Orientation adaptation can be shown physiologically by prolonged exposure to an oriented stimulus reducing firing rates of responsive orientation-selective cells [10]–[11] and psychophysically by exposure to high-contrast gratings reducing either the visibility or perceived contrast of subsequently presented low-contrast gratings [12]–[20]. Both approaches reveal orientation tuning: as the angular difference between target and adaptor or mask increases, adaptation- and masking-induced threshold elevation decreases.

Both psychophysically and physiologically derived estimates of orientation selectivity (bandwidth) are highly variable. Even within the primary visual cortex (V1) of individual macaques (a widely cited homologue of the human visual system) bandwidth estimates range from very narrow (6° half-width at half amplitude (HWHA)) to completely untuned [21]–[22]. Psychophysical estimates based on masking and adaptation also show substantial variability, with estimates ranging from ∼12°–40° HWHA in masking studies [9], [23]–[24] to between ∼7° [16] and ∼100° for adaptation [16], [20], [25]. One factor believed to account for these variations in orientation bandwidth is spatial frequency, with tunings narrowing as frequency increases. Over the range tested psychophysically, orientation bandwidths have been estimated to be up to three times broader at low, relative to high, spatial frequencies, regardless of whether derived from masking or adaptation. Indeed, meta-analysis of the psychophysical masking and adaptation literature yields a strong negative correlation between spatial frequency and orientation bandwidth [8].

A possible explanation for the covariance of bandwidth and spatial frequency comes from recent psychophysical studies finding evidence for two distinct components of orientation masking: an orientation-tuned component and an un-tuned (or far more broadly tuned) suppressive component [5], [8], [26]–[27]. Since early masking-derived estimates were based on models containing tuned but not untuned parameters, these estimates of orientation-tuning may have conflated the two components, thereby accounting for the considerable variability in bandwidth estimates with spatial frequency [9], [20]. Two psychophysical studies [8], [28] confirmed this recently using an overlay masking paradigm. Both found bandwidths to be remarkably stable (∼20–30° HWHA) across all regions of spatio-temporal frequency space when fitted with Gaussian functions comprising an additional ‘untuned’ (orientationally isotropic) component (which is effectively a baseline elevation). Critically, when fitted without this isotropic parameter, bandwidth estimates were significantly broader below 2 c.p.d.. This suggests that the reported dependency of orientation-bandwidth on spatial frequency may be spurious and largely a consequence of covariation in the amplitude of isotropic threshold elevation (due to masking) with increasing spatial frequency [8].

The idea that masking is a consequence of both orientation-tuned and -untuned factors receives convergent physiological support from the phenomenon known as cross-orientation masking (also known as cross-orientation suppression). Cross-orientation masking describes the observation that the response of an orientation-selective V1 neuron to an optimally oriented stimulus presented in isolation, may be suppressed by a spatio-temporally superimposed stimulus whose orientation exceeds the bandwidth of the cell's classical receptive field [29]–[37]. Although cross-orientation masking was initially thought to occur only in response to orthogonal masks, more recent evidence indicates that cross-orientation masking occurs in response to superimposed masking stimuli of any orientation (i.e., it is isotropic) [33], [35]. Although both psychophysical and physiological results provide strong support for the inclusion of an isotropic parameter in masking-derived estimates of orientation bandwidth, the neural mechanism(s) of isotropic masking remain a matter of considerable debate [29], [35], [38]–[39], with some favouring a pre-cortical and others a cortical locus.

Our primary aim in this study is to establish whether there is evidence for isotropic adaptation, and if so, to what extent it depends upon spatial frequency. If we do find evidence of isotropic adaptation, a second aim is to establish whether adaptation-derived orientation-specific (bandwidth) effects vary as a function of spatial frequency following the inclusion of the isotropic parameter into our fits. A third aim is to establish whether isotropic adaptation transfers dichoptically. Psychophysical isotropic masking effects exhibit strong interocular transfer [5], [8], suggesting a cortical locus. If psychophysical isotropic adaptation effects are also mediated at a cortical locus, we would expect to observe them under conditions in which adaptor and target are presented to the same eye (monoptically) and to different eyes (dichoptically). By contrast, if psychophysical isotropic adaptation effects are mediated at purely pre-cortical loci we would expect them to be limited to monoptic rather than dichoptic adaptation conditions.

## Materials and Methods

### Ethics statement

Written consent was obtained from each participant prior to the experiment. The experiment was approved by the local ethics committee of the University of Sydney.

### Participants

The participants were five adults with normal or corrected-to-normal vision (age 21–43 years, mean = 29 years). All were right-eye dominant. Three participants were experienced psychophysical observers. Two participants (AJ and JC) were aware of the hypotheses, whereas the others were naïve to the purposes of the study. All participants were trained for at least one half-hour session before testing commenced.

### Apparatus

The experiment was programmed with MATLAB version 7.4, using the Psychophysics Toolbox version 3 [40]. Stimuli were presented using an ATI Radeon ×1600 graphics card driving a linearised CRT monitor with a screen resolution of 1024 by 768 pixels and a refresh rate of 100 Hz. 10.8 bit luminance resolution was achieved using a bit-stealing algorithm. Participants viewed the screen through a bench-mounted mirror Wheatstone stereoscope from a total path length of 57 cm, and made their responses on four separate keys mounted on a numerical keypad.

### Design

We systematically manipulated the following stimulus variables:

- Angular difference between adaptor and test: 0°, 22.5°, 45°, 67.5°, 90°, −22.5°, −45° and −67.5°). A horizontal and a vertical test stimulus were presented on every trial. Two values of angular difference separated by 90° were presented in each block of trials.
- *Spatial frequency*. 0.25 and 2 c.p.d., blocked across trials; and
- *Ocular presentation mode*. Retinotopically aligned adaptor and test presented to the same eye (Monoptic); or different eyes (Dichoptic). Monoptic and dichoptic conditions were randomly interleaved across trials.

### Stimuli

All stimuli were sine-wave gratings presented within circular windows with a diameter of 4° visual angle and a cosine ramped outer edge (λ = 10 pixels). Stimuli were presented on a grey background with mean luminance of 52 cd/m^2^. The spatial frequency of both the adaptor and test stimuli was fixed at either 0.25 or 2 c.p.d.. These frequencies were used because Cass et al. (2009) found the greatest difference in isotropic masking effects at similar spatial frequencies: 0.5 and 2 c.p.d.. Stimuli were sinusoidally counterphase modulated at a temporal frequency of 10 Hz in all conditions.

Eight adaptor orientations were employed: 0°, 22.5°, 45°, 67.5°, 90°, −22.5°, −45° and −67.5°. Each adaptor orientation was paired with two test orientations: one vertical (0°) and one horizontal (90°), creating 16 adapt-test pairings. On any given block of trials, two adaptor orientations were used, each differing by 90°. The paired adaptor and test orientations resulted in eight levels of angular difference between adaptor and test: 0°, 22.5°, 45°, 67.5°, 90°, −22.5°, −45° and −67.5°. The term ‘angular difference’ refers to the positive or negative angle (for the right and left sides of the orientation tuning curve, respectively) between the adaptor and test stimuli. This allowed data to be collected across 180°, a full orientation tuning function symmetrical around 0° angular difference. Each level of angular difference was presented both monoptically and dichoptically. This created a total of 32 adapt-test pairings. The relative locations and eye of presentation for each adaptor orientation were preserved across trials, but randomised across testing blocks.

Table 1 outlines how the 32 adapt-test pairings were grouped into 16 conditions, according to angular difference between adaptor and test stimuli, and ocular presentation mode. Each condition contained two equivalent adapt-test pairings: one with a vertical test (0°) and the other with a horizontal test (90°). The data obtained using vertical and horizontal tests were pooled, effectively doubling the amount of data for each level of angular difference. For each given block of trials, each of the eight possible stimulus configurations (see Table 1) was randomised across trials.

**Table 1:** 32 adapt-test pairings, grouped into sixteen conditions according to angular difference and ocular presentation mode.

Block	Ocular presentation mode	Adaptor Orientation (°)	Test Orientation (°)	Angular Difference (°)
1	Monoptic	0	0	0
		90	90	
		−90	0	90
		−0	90	
	Dichoptic	0	0	0
		90	90	
		−90	0	90
		−0	90	
2	Monoptic	22.5	0	22.5
		67.5	90	
		−67.5	0	−67.5
		−22.5	90	
	Dichoptic	22.5	0	22.5
		67.5	90	
		−67.5	0	−67.5
		−22.5	90	
3	Monoptic	45	0	45
		45	90	
		−45	0	−45
		−45	90	
	Dichoptic	45	0	45
		45	90	
		−45	0	−45
		−45	90	
4	Monoptic	67.5	0	67.5
		22.5	90	
		−22.5	0	−22.5
		−67.5	90	
	Dichoptic	67.5	0	67.5
		22.5	90	
		−22.5	0	−22.5
		−67.5	90	

Each adaptor orientation appears four times, paired with a vertical test (0°) and a horizontal test (90°), and each adapt-test pairing is presented monoptically and dichoptically.

**Figure 1:**
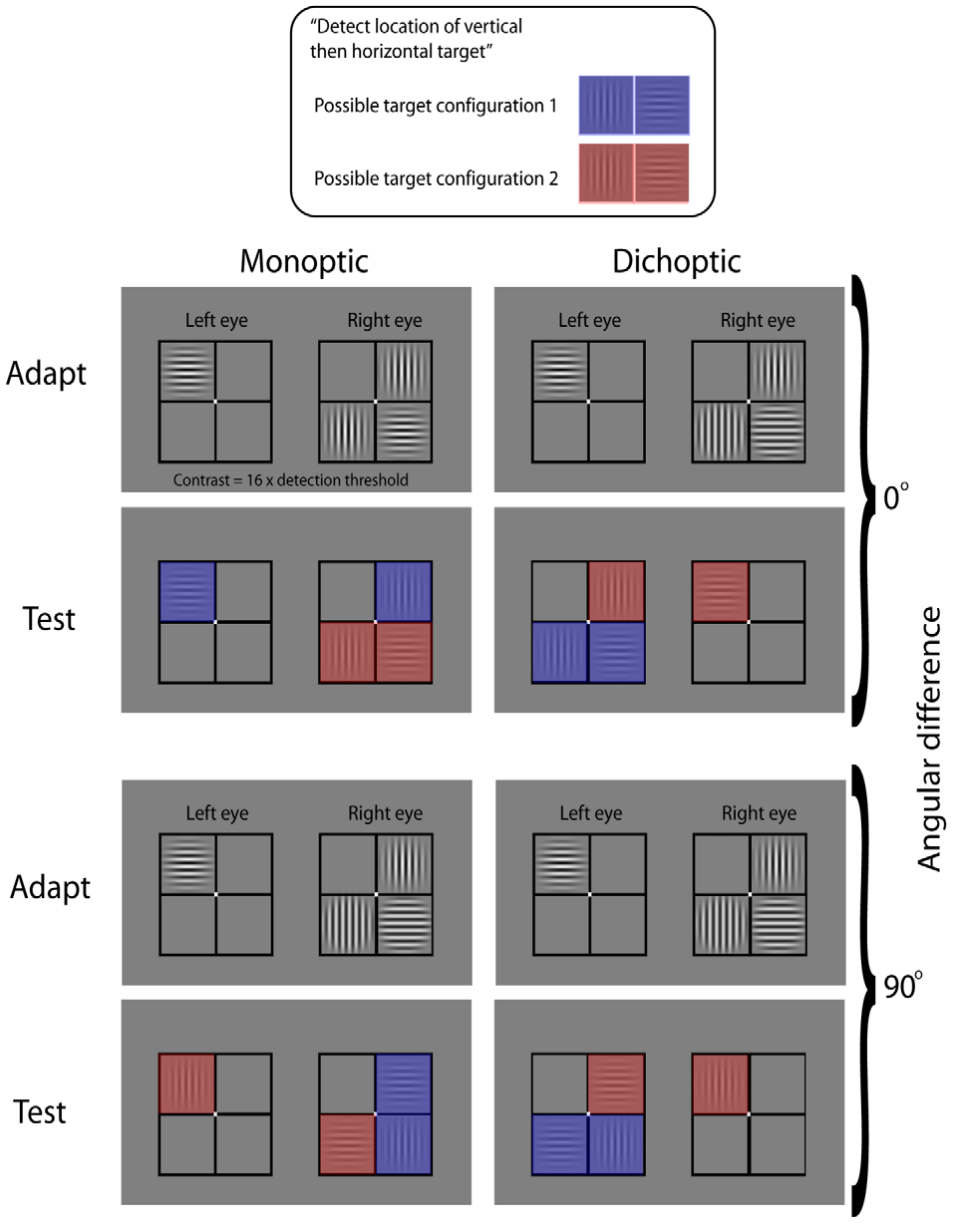
An example of the spatial configuration of adaptor and test stimuli. Four conditions are shown (clockwise from left): monoptic 0°, dichoptic 0°, dichoptic 90° and monoptic 90°. The figure provides an example of possible adaptor stimuli configurations, and the two possible configurations of target stimuli.

Figure 1 provides an example of the spatial configuration of the adaptor and test stimuli within a single block of trials (in this case describing angular differences = 0° and 90°). The display consisted of two sets of four fusion locking squares, each with dimensions of 4°×4° of visual angle, with lines of 1 pixel in width. One set of four fusion locking squares was presented to the left eye, and another set of four squares presented to the right eye. Each set of four squares surrounded a central fixation point of 0.3° of visual angle. Given the fusion locking, the subject perceived one fixation point surrounded by four squares. There were a total of eight possible stimulus locations (four in the left and four in the right eye).

Adaptor gratings appeared in four of the eight locations: two gratings at one orientation (for example, 0°) and two gratings at the orthogonal orientation (for example, 90°), each presented to either the left or the right eye. The four adaptor stimuli occupied each of the four possible perceived visual locations (see Figure 1). The positions of the adaptor stimuli remained constant within each testing block, but were varied across testing blocks. The two test stimuli, one vertical and one horizontal, were presented in two of the eight possible locations. Each test stimulus was positioned so that it was paired with an adaptor stimulus according to the conditions outlined in Table 1. Observers viewed the display through the stereoscope and the mirrors were adjusted until the two sets of four fusion-locking squares (presented separately to each eye) were superimposed perceptually. Observers were unaware of the eye to which the stimuli were presented within any given block.

### Procedure

Subjects were instructed to identify the locations of the two simultaneously presented target gratings. This required two separate responses: the location of the vertical, followed by the location of the horizontal test grating. This task was conducted both prior to (i.e., baseline condition) and following prolonged exposure to four high-contrast gratings (16× threshold) of variable orientation (adaptation conditions). On the first trial within any given block, the period of adaptation was 20 seconds, followed by 5 seconds of ‘top-up’ adaptation on subsequent trials. The two test stimuli (one vertical and one horizontal) were then presented simultaneously for 640 milliseconds in two of the four perceived locations. Subjects were required to first identify the location of the vertical target, then the location of the horizontal target. The task was a spatial four-alternative forced-choice (4AFC) task with binary orientation identification adapted from [41]–[42]. Responses were made using a standard configuration number keypad. Keys 1 and 4 corresponded to locations left of fixation in lower and upper visual fields respectively, with keys 2 and 5 corresponding locations to the right of fixation in lower and upper visual fields respectively. The chance level was 25% for the first response, followed by 31.6% for the second response [41], corresponding to detection rates of 25% on the 21% of trials where the first target was not detected, plus 33% on the 79.4% trials where the first target was detected, and produced an overall chance performance rate of 28% across conditions. Thus our psychometric functions were constrained to have a lower asymptote of 28%, an upper asymptote of 100%, with threshold defined as contrast yielding 64% correct detection performance.

Visual feedback was provided via the bipartite fixation point: white for a correct response and black for an incorrect response. The colour of the left side of the fixation point corresponded to the vertical response (first response), the right side to the horizontal response (second response). This task has two distinct advantages over the two-alternative forced-choice (2AFC) detection tasks used in many adaptation studies. The first is that the task improves efficiency, requiring responses to two adapt-test pairings in each trial. Second, there is evidence for the existence of two channels in spatio-temporal vision: one that detects the spatial properties of the stimuli and one that detects transient movement or flicker [43]–[45]. The threshold for detecting movement of transient stimuli, however, is typically lower than the threshold for identifying spatial structure, particularly at low spatial frequencies. A task that relies on detection of the stimuli, such as a 2AFC task, may measure the threshold for visual transients rather than spatial structure, and may therefore underestimate thresholds differentially across spatial frequencies. The 4AFC task used in the present study should provide more accurate threshold estimates for pattern rather than transient detection, as it requires the participant to identify the spatial orientation (i.e., the pattern) of the test stimuli rather than relying on its transient onset or offset [41].

The pairing of stimulus orientation and ocular mode of presentation is outlined in Table 1. The adapt-test pairings were grouped into four blocks of trials. Each block of trials tested two levels of angular difference, for example, 0° and 90°, and tested four conditions, for example, 0° monoptic, 90° monoptic, 0° dichoptic and 90° dichoptic (see Figure 1 for an example of stimulus spatial configuration). Each of the four conditions was tested by an independent trial, and the trials were randomly interleaved throughout each block. Each trial consisted of two adapt-test pairings. A total of 160 responses were therefore made in each block: four conditions, with two adapt-test pairings in each, and 20 responses for each pairing.

### Threshold analysis

For all conditions, contrast detection thresholds were estimated using an adaptive staircase procedure. Eight separate staircases were used in each block, one for each of the adapt-test pairings outlined in Table 1. The staircase controlled the root mean squared contrast of the target following a 3-down, 1-up rule which converges on 79.4% correct performance [46]. The staircases were initialized with starting contrasts 4 dB above and below the thresholds estimated in pilot runs. The step size was initially 1 dB and reduced to 0.5 dB after 4 reversals, and the staircase terminated after 30 responses.

## Results

Estimates of threshold were obtained by pooling each subject's horizontal and vertical test data across testing blocks for each experimental condition (angular difference, spatial frequency and ocular mode of presentation). Data from at least four separate staircase runs were combined at each level of adaptor-test angular difference and fit with a cumulative Gaussian function by weighted minimization of chi-square (performance at each contrast level was weighted by the binomial standard deviation based on the number of trials presented at that contrast). The lower asymptote was 28% correct performance (the overall chance rate) and the contrast predicting 64% correct performance (the midpoint between 28% and 100%) defined the detection threshold estimate [41]. Ninety-five percent confidence intervals on this point were calculated with a bootstrapping procedure based on 500 data sets simulated from the number of experimental trials at each level tested [47].

**Figure 2:**
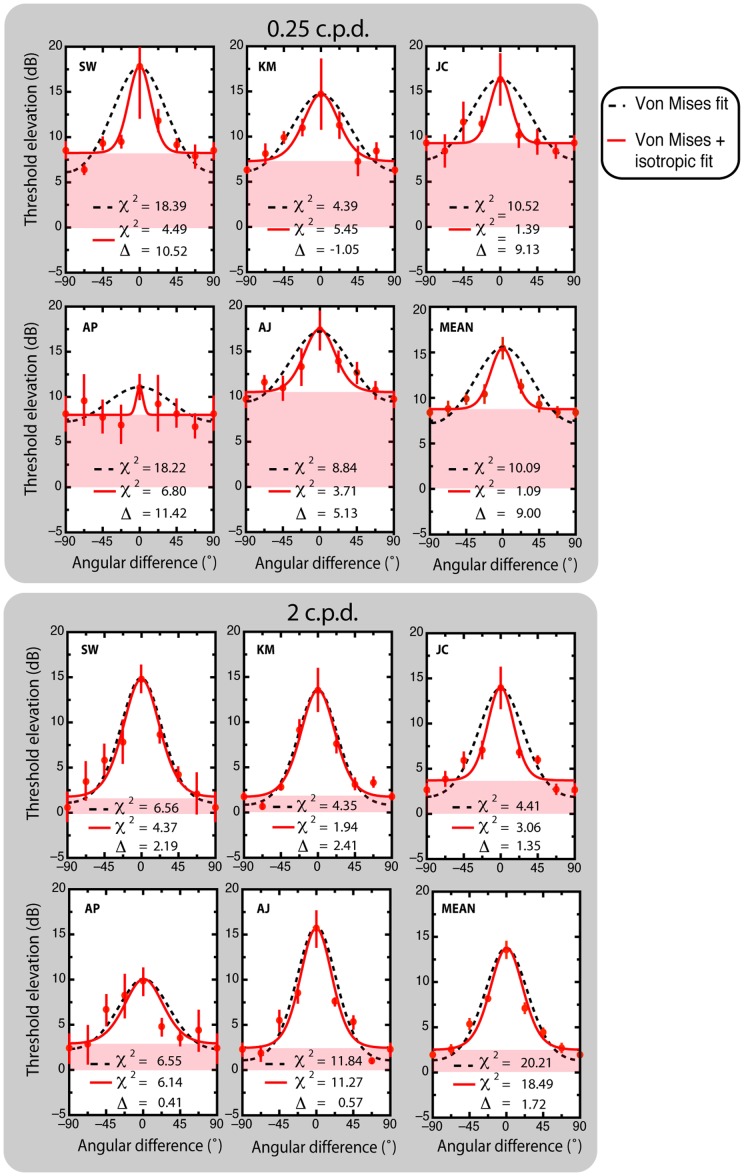
Individual and mean monoptic threshold elevation estimates as a function of angular difference between adaptor and test stimuli. The data points are fitted with a standard von Mises model (Equation 1; black dashed lines) and a von Mises model with an isotropic amplitude component included (Equation 2; solid lines). Chi-square estimates of each fit are included for individual subjects and averaged data. Differences in chi-square greater than 3.84 are deemed statistically significant (*p*<.05, 1 degree of freedom).

**Figure 3:**
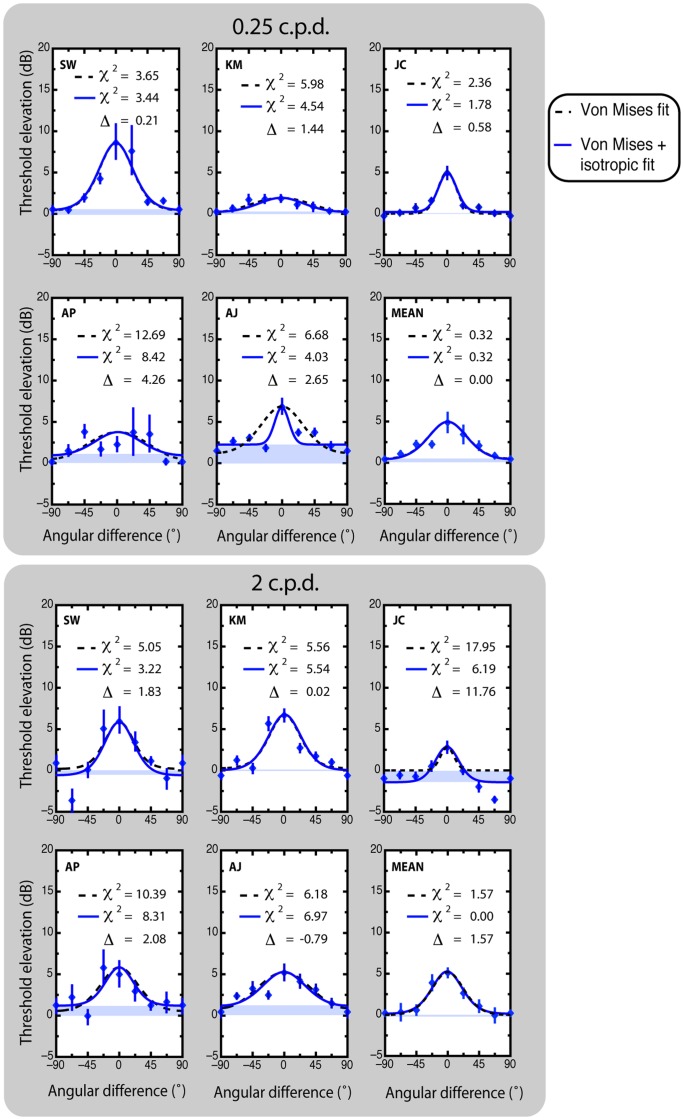
Individual and mean dichoptic threshold elevation estimates as a function of angular difference between adaptor and test stimuli. The data points are fitted with a standard von Mises model (Equation 1; black dashed lines) and a von Mises model with an additive isotropic amplitude component (Equation 2; solid lines). Chi-square estimates of each fit are included for individual subjects and averaged data. Differences in chi-square greater than 3.84 are deemed statistically significant (*p*<.05, 1 degree of freedom).

Unadapted (baseline) and adapted threshold estimates were measured in separate blocks for the different spatial frequencies, and the ratio of adapted to unadapted threshold was used to compute threshold elevation (O), measured in decibels (dB); where O_dB_ = 20×log10 (adapted threshold/unadapted threshold). Elevations in threshold were then plotted as a function of angular difference between target and adaptor stimuli for each spatial frequency (0.25 and 2 c.p.d.) and ocular mode of presentation (monoptic and dichoptic) (Figures 2 and 3 respectively).

### Isotropic effects

Previous psychophysical studies of orientation channels have tended to implement Gaussian models. Orientation, however, is a periodic dimension, whereas Gaussian models are non-periodic. A solution to this has been the implementation of models which characterise circular variance, the most popular of which is the von Mises model. Each subject's data were fitted with a von Mises function to characterise the peak and bandwidth of orientation tuning. Two forms of the von Mises function were compared. The first had the form:
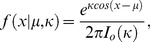
(1)where *I*_0_(*x*) is the modified Bessel function of order 0, x = angular difference between adaptor and test stimuli, *κ* = circular variance (deformation parameter), and *μ* = peak angular difference. *μ* was fixed at 0° whilst *κ* was free to vary.

The second von Mises function was identical in all respects but contained an additional free parameter, α, which is a baseline elevation describing an isotropic (i.e. untuned) adaptation component.
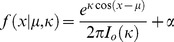
(2)Thus a total of eight independent orientation tuning functions were fitted for each subject: monoptic (Figure 2) and dichoptic (Figure 3), both at 0.25 and 2 c.p.d., each fitted with and without the isotropic parameter.

To determine whether the addition of the isotropic parameter (α) produced a significant improvement in fit we compared chi-square estimates for each fit on a subject-by-subject basis. The chi-square value quantifying the goodness of fit between the data points and the fitted von Mises function is given by the following formula:
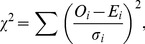
(3)where *O_i_* = observed threshold elevation for a particular angular difference between adaptor and test (indexed by subscript *i*), *E_i_* = is the value of the fitted von Mises function at point *i*, and *σ_i_* = standard deviation of the observed threshold elevation at point *i* (as determined by bootstrapping).

**Figure 4:**
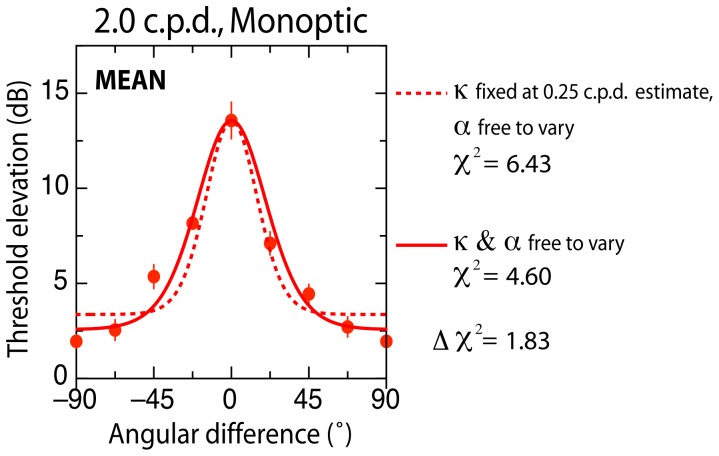
Comparison of circular variance (κ) as a function of spatial frequency. Data represent threshold elevation averaged across subjects in the 2 c.p.d. monoptic condition. In one case, Equation 2 is fit with two free parameters (κ and α; solid curve) while in the other it is fit with only one free parameter (α), with κ was fixed at the value found in the 0.25 c.p.d. monoptic condition (dashed curve). The difference between these fits fails to exceed the critical value of 3.84 (p>.05; 1 degree of freedom), indicating no significant difference in bandwidths (circular variance) across spatial frequency.

The two von Mises functions are identical in all ways but for a single additive isotropic parameter (α) in the second equation. Equation 1 is therefore a nested version of Equation 2. The statistical significance of this single parameter can be ascertained by first calculating the chi-square values associated with the residual error of each fit. The difference between these chi-square estimates (χ^2^_diff_) can then be compared to the critical chi-square value associated with a single degree of freedom (χ^2^ = 3.84 for alpha = 0.05). To demonstrate that Equation 2, with its extra parameter, produces a statistically better fit than Equation 1, the difference between the chi-squares for the two von Mises fits (χ^2^_1_ minus χ^2^_2_) must exceed this critical value. Otherwise, we accept the null hypothesis that Equations 2 does not produce a better fit than Equation 1. Fitted free parameter and chi-square estimates for each fit, subject and condition are detailed in Table 2. Chi-square estimates and nested model comparisons are also included in Figures 3 & 4.

**Table 2:** Parametric, bandwidth and chi-square and estimates for each spatial frequency and ocular mode of presentation using Von Mises Equations 1 and 2.

Condition	Subject	vmMin	HH	HWHH	κ	α	r^2^	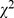	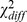 Eq.1–Eq.2	Model
**0.25 c.p.d. MON**	AJ	0.54	0.77	40.62	0.31		0.83	8.84		Eq. 1
		0.61	0.80	12.60	2.29	0.85	0.89	3.71	5.13*	Eq. 2
	AJP	0.67	0.83	42.09	0.20		0.14	18.22		1
		0.73	0.87	2.19	49.11	7.61	0.53	6.80	11.42*	2
	SW	0.34	0.67	37.47	0.54		0.66	18.39		1
		0.46	0.73	10.71	4.54	0.71	0.91	4.49	13.90*	2
	JC	0.47	0.73	39.65	0.38		0.61	10.52		1
		0.57	0.79	8.72	5.21	1.19	0.84	1.39	9.13*	2
	KM	0.42	0.71	38.95	0.43		0.84	4.39		1
		0.49	0.75	16.27	1.86	0.46	0.88	5.45	−1.05	2
	**MEAN**	**0.48**	**0.74**	**39.90**	**0.36**		**0.74**	**10.09**		**1**
		**0.58**	**0.79**	**10.02**	**3.89**	**1.05**	**0.95**	**1.09**	8.99*	**2**
**2 c.p.d. MON**	AJ	0.07	0.53	28.88	1.35		0.92	11.84		1
		0.15	0.58	19.95	2.36	0.10	0.93	11.27	0.57	2
	AJP	0.25	0.63	35.67	0.69		0.73	6.55		1
		0.31	0.66	22.21	1.47	0.16	0.73	6.14	0.41	2
	SW	0.08	0.54	29.41	1.28		0.93	6.56		1
		0.13	0.56	23.73	1.78	0.05	0.93	4.37	2.19	2
	JC	0.13	0.57	32.11	1.00		0.84	4.41		1
		0.27	0.63	15.02	3.40	0.25	0.89	3.06	1.35	2
	KM	0.06	0.53	28.54	1.39		0.95	4.35		1
		0.13	0.56	22.40	1.97	0.06	0.97	1.94	2.41	2
	**MEAN**	**0.11**	**0.56**	**31.24**	**1.09**		**0.94**	**20.21**		**1**
		**0.20**	**0.60**	**20.46**	**2.10**	**0.12**	**0.95**	**18.49**	**1.72**	**2**
**0.25 c.p.d. DICH**	AJ	0.19	0.59	33.89	0.54		0.84	6.68		1
		0.36	0.68	8.70	8.52	0.63	0.74	4.03	2.65	2
	AJP	0.28	0.64	36.33	0.64		**0.33**	12.69		1
		0.16	0.58	45.23	0.00	−0.16	**0.45**	8.42	4.26*	2
	SW	0.06	0.53	28.22	1.42		0.91	3.65		1
		0.06	0.53	27.55	1.47	0.01	0.91	3.44	0.21	2
	JC	0.00	0.50	16.54	4.27		0.94	2.36		1
		0.03	0.52	15.31	4.73	0.03	0.94	1.78	0.58	2
	KM	0.26	0.63	35.78	0.68		0.72	5.98		1
		0.16	0.58	42.33	0.00	−0.16	0.78	4.54	1.44	2
	**MEAN**	**0.01**	**0.51**	**23.28**	**2.18**		**0.95**	**0.32**		**1**
		**−0.04**	**0.48**	**26.19**	**1.87**	**−0.03**	**0.96**	**0.32**	**0.00**	**2**
**2 c.p.d. DICH**	AJ	0.23	0.62	35.14	0.73		0.73	6.18		1
		0.18	0.59	41.39	0.24	−0.11	0.76	6.97	−0.79	2
	AJP	0.11	0.56	31.14	1.10		0.62	10.39		1
		0.21	0.60	20.44	2.06	0.13	0.66	8.31	2.08	2
	SW	0.02	0.51	24.42	1.97		0.74	5.05		1
		−0.12	0.44	34.04	1.31	−0.07	0.75	3.22	1.83	2
	JC	0.00	0.50	11.57	8.61		0.60	17.95		1
		−0.55	0.22	29.50	3.09	−0.24	0.72	6.19	11.76*	2
	KM	0.03	0.52	26.15	1.70		0.85	5.56		1
		0.01	0.50	28.42	1.51	−0.02	0.85	5.54	0.03	2
	**MEAN**	**0.01**	**0.51**	**23.28**	**2.18**		**0.95**	**0.32**		**1**
		**−0.04**	**0.48**	**26.19**	**1.87**	**−0.03**	**0.96**	**0.32**	**0.00**	**2**

Note, higher estimates of circular variance (κ) indicate narrower orientation bandwidths. Asterisks indicate that chi-square estimates derived from Eq. 1 and 2 exceed χ^2^_critical_ = 3.84.

As can be seen in Table 2 and Figures 3 & 4, differences in chi-square estimates (χ^2^_diff_) associated with the two fits failed to exceed χ^2^_critical_ for any subject in the 2 c.p.d. conditions (monoptic and dichoptic), nor for the 0.25 c.p.d. dichoptic condition. However, in the 0.25 c.p.d. monoptic condition, χ^2^_diff_ exceeded the critical value for four out of five subjects. The pattern of data was qualitatively similar in the averaged data.

### Bandwidths

In the Von Mises model, the circular variance parameter, κ, governs the rate at which threshold elevation varies as a function of angular difference between adaptor and test orientations. Table 2 shows that including the isotropic parameter (α) increases κ for all subjects in the 0.25 c.p.d. monoptic condition. For the averaged data, including the isotropic parameter increases estimates of κ by more than a factor of ten (κ = 0.36 vs 3.89), meaning narrower tuning bandwidths (see upper half of Figure 2). For the 2 c.p.d. conditions there was no significant change in κ when the isotropic parameter was included.

Classically, orientation bandwidths are expressed in degrees, typically HWHA. HWHA estimates were derived from each von Mises function by finding the angular difference producing half-maximum threshold elevation (maximum amplitude was at 0° angular difference, the minimum was taken as the minimum value of the fitted function). For the monoptic 0.25 c.p.d. condition, which was better fit by the von Mises function with an isotropic component (Equation 2), a paired t-test reveals that the better fit is accompanied by a significant decrease in orientation bandwidth relative to bandwidths without an isotropic component (M = 39.7° vs 10.10°; t_4_ = 10.26, *p* = .001). The fits obtained from subject AP in this condition are very poor (r^2^ = .14 & .53). Subject KM failed to show a statistically significant difference in fit. We reanalyzed the effect of fit type on HWHA with AP's and KM's data omitted from the paired subjects t-test. The effect remains significant (M = 39.2° vs 10.7°; t_2_ = 23.11, *p*<.01).

### Effects of spatial frequency on bandwidths

The question of whether different spatial frequencies yield different orientation bandwidths can be answered by comparing whether the factor governing bandwidths (κ) explains a statistically greater proportion of the variance across spatial frequency than that which is unrelated to bandwidth (α). To determine whether this is the case we compare chi-square estimates for the 2 c.p.d. monoptic condition using Equation 2. In one case, Equation 2 is fit with two free parameters (κ and α) while in the other it is fit with only one free parameter (α). To make Equation 2 a one-parameter model, the value of κ was fixed at the value found in the 0.25 c.p.d. monoptic condition.

The results of this comparison indicate that despite the higher spatial frequency condition yielding smaller estimates of HWHA (see Table 2), chi-square estimates derived from fits to the averaged data obtained in the 2 c.p.d. monoptic condition when α and κ are free to vary (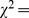
4.60) are not significantly different from those derived from fits in which α alone is free to vary (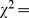
6.43) (χ^2^_diff_ = 1.83; χ^2^ = 3.84) (see Figure 4). Similarly, analysing individual subjects, chi-square differences were not statistically significant in four out of five observers (AJ: 0.12; AP: 2.91; JC: 0.21; KM: 0.09), with only one observer yielding a critical difference (SW: 4.31). In general, then, the isotropic parameter (α) alone can account for differences in elevation observed across the tested spatial frequencies in the monoptic condition, and implies that this variance can be explained without allowing the circular variance parameter (κ) to vary freely. Given that the circular variance parameter governs bandwidth, we conclude that orientation bandwidths do not vary significantly across the spatial frequencies tested.

### Effects of ocularity on bandwidths

**Figure 5:**
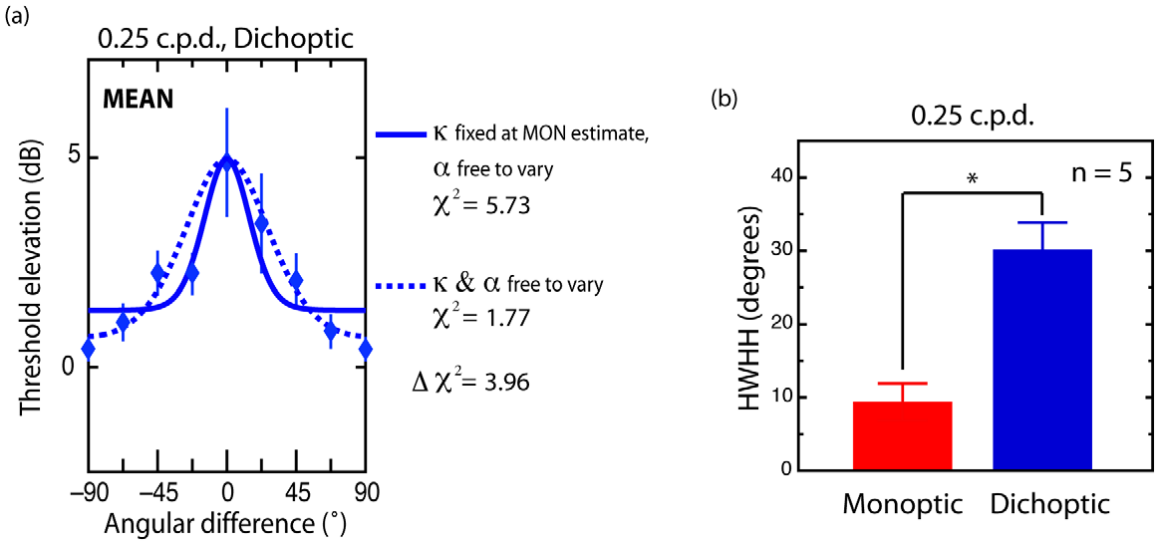
Effect of ocular presentation mode on bandwidths. (a) Comparison of circular variance (κ) as a function of ocular presentation mode. Data represent threshold elevation averaged across subjects in the 0.25 c.p.d. dichoptic condition. In one case, Equation 2 is fit with two free parameters (κ and α; solid curve) while in the other it is fit with only one free parameter (α), with κ was fixed at the value found in the 0.25 c.p.d. monoptic condition (dashed curve). The difference between these fits exceeds the critical value of 3.84 (p>.05; 1 degree of freedom), indicating broader bandwidths (smaller estimates of κ) in the dichoptic condition. (b) Average estimates of bandwidth (HWHA) measured under monoptic and dichoptic adaptation conditions (0.25 c.p.d.) using Equation 1 for the dichoptic and Equation 2 (κ and α both free to vary) for the monoptic condition.

Chi-square estimates derived from fits of the averaged data obtained in the 0.25 c.p.d. dichoptic condition when α and κ are free to vary (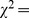
1.77), are significantly smaller (though marginally) from those derived from fits in which α alone is free to vary (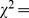
5.73), with κ fixed at the value observed in the 0.25 c.p.d. monoptic condition (χ^2^_diff_ = 3.96; χ^2^ = 3.84) (see Figure 5a). This was reflected in individual analyses: significantly smaller chi-square values were observed in the fits of four out of the five individual subjects (AJ: 4.79; AP: 13.65; SW: 6.63; KM: 9.29), with subject JC not significant (0.18). These results show that in general the isotropic parameter (α) cannot on its own account for differences in the pattern of elevation observed between monoptic and dichoptic conditions and implies that the circular variance parameter (κ) accounts for a significant proportion of this variance. Given that the circular variance parameter governs bandwidth, we conclude that orientation bandwidths are significantly broader under conditions of dichoptic compared with monoptic adaptation. This is further confirmed by a paired t-test comparing 0.25 c.p.d. monoptic and dichoptic estimates of HWHA (t_4_ = −4.75, *p*<.01) (10.1° vs 30.2°) (see Figure 5b).

## Discussion

The present study examined the orientation-dependence of contrast adaptation expressed as elevation in grating detection thresholds measured as a function of the angular difference between adaptor and test stimuli. The adaptation-induced threshold elevations were measured at two spatial frequencies (0.25 and 2 c.p.d.) under monoptic and dichoptic conditions. All threshold elevations exhibit strong orientation tuning and are well described by Von Mises models of circular variance in the orientation domain (Equation 1). A significant improvement in fit was observed in the 0.25 c.p.d. monoptic condition when an orientation non-specific parameter was added (Equation 2). Critically, this improvement in fit did not extend to the higher spatial frequency or dichoptic conditions.

When fitting our monoptic data with standard Von Mises fits (Equation 1) we observe the common finding of narrower orientation bandwidths (greater circular variance) with increasing spatial frequency (∼40°–30° HWHA) [9], [20]. This effect of spatial frequency on orientation bandwidths, however, became non-significant with the addition of the isotropic parameter to the fits. The isotropic parameter's stabilising effect on orientation bandwidths is reminiscent of two recent masking studies [8], [28]. In those studies, orientation bandwidths were estimated to be approximately 20°–30° HWHA, generally broader than those observed here (∼10°–20° HWHA) at comparable spatial frequencies. That both masking and adaptation paradigms now demonstrate that orientation bandwidths do not vary significantly across spatial frequency suggests that previous studies showing decreasing bandwidths with increasing spatial frequency may have confounded cross-orientation adaptation (or masking) with bandwidth. Why our adaptation paradigm should yield narrower estimates of bandwidth than the masking studies of [8] or [28] is not clear. Recently [5] confirmed an earlier observation by [26] that orientation-tuned masking effects may themselves be composed of two separate components: one being a narrowband, phase-sensitive summative process; and the other a broader, phase insensitive mechanism (∼7°–30° HWHA, respectively). We speculate that the narrow bandwidths we observe using adaptation could be due to relatively greater adaptability of the narrower summative component.

Despite finding no differences in bandwidth as a function of spatial frequency (after correcting for isotropic adaptation effects), we did observe differences based on ocularity, with interocular adaptation producing broader bandwidths at the lower spatial frequency (see Figure 5b). Although we made no predictions about such broadening, its presence suggests that the interocular interactions mediating orientation-selective adaptation may be noisier than those involved within monocular channels. A similar broadening of orientation selectivity has recently been observed in the context of binocular rivalry [48] and this was also attributed to an increase in interocular noise. This observation is interesting in light of contemporary models of rivalry which postulate that interocular adaptation and rivalry share common processes [49]–[51].

With respect to the untuned adaptation component, we found that the inclusion of the isotropic parameter significantly improved our Von Mises fits in only one out of the four conditions tested (0.25 c.p.d. monoptic adaptation). Not only was there no improvement in fit at the higher spatial frequency tested (2 c.p.d.), we were surprised to find no evidence of untuned adaptation in the dichoptic condition [52]. A similar set of stimulus contingencies was recently published whilst this paper was under review [53]. Because binocularity and orientation-selectivity are strongly evident in the primate visual hierarchy until area V1 [2]–[3], the isotropic adaptation we observe could conceivably occur at a level preceding the emergence of orientation-selectivity, or possibly independently of it. Strong adaptation (20 Hz modulation) that is both isotropic and entirely monocular has been reported in magnocellular layers of primate lateral geniculate nucleus [54], in response to similarly fast rates of temporal modulation to those employed in this study. One must also consider the possibility that isotropic adaptation may be mediated either at, or within, the first synapses subsequent to the thalamo-cortical interface, prior to binocular integration. Alternatively, they may arise from non-specific inhibitory effects between orientation-selective neurons [34], although these would need to be monocularly driven, constraining their locus to the earliest cortical laminae in V1 (possibly layer IVc), or if occurring later, to within ocular dominance columns.

Interestingly, whereas isotropic adaptation is limited to situations in which the same eye is adapted and tested, psychophysical cross-orientation masking exhibits robust (if not complete [55]) interocular transfer [8]. Physiological evidence for interocular transfer of cross-orientation masking is more equivocal, with some studies showing it is weak [56]–[58] or absent [33], [59], whilst others find substantial interocular transfer [60]–[61]. Despite the complete interocular transfer of cross-orientation masking observed by [8], monoptic and dichoptic cross-orientation masking may nonetheless be mediated by different mechanisms. Indeed [55], showed that monoptic and dichoptic cross-orientation masking differentially depend upon the spatio-temporal frequency properties of the stimulus, with dichoptic cross-orientation masking generally stronger than monoptic conditions at higher spatial and lower temporal frequencies (4 vs 15 Hz). Why the cross-orientation adaptation observed in the current study should be restricted to monocular conditions, rather than the substantial transfer observed with cross-orientation masking [8], [55], is unknown.

One possibility is that if dichoptic cross-orientation masking is a consequence of inhibitory interactions between monocular (or ocularly-biased) neurons of the kind assumed in reciprocal interocular inhibition models of binocular rivalry [49], then adapting these inhibitory interactions may result in their disinhibition [62]. Conceivably, such disinhibition would predict little or no threshold elevation, or possibly even threshold reduction (i.e., facilitation). Whether the absence of dichoptic adaptation in our results reflects interocular disinhibition is unclear, although we do note a small amount of untuned facilitation at the higher spatial frequency tested (2 c.p.d.). Future research involving systematic manipulation of adaptor contrast and periods of adaptation may shed light on this hypothesis.

A curious feature of these data is that the magnitude of orientation-specific adaptation dissociable from that of isotropic adaptation with respect to both spatial frequency and ocular mode of presentation. For example, whereas under monoptic adaptation conditions isotropic adaptation is only evident at the lower spatial frequency tested, amplitude of orientation-specific adaptation is greater at the higher spatial frequency. Curiously, neither of these spatial frequency effects are evident under dichoptic conditions. That these spatial frequency effects should fail to transfer between the eyes points to both orientation-specific and non-specific monoptic adaptation being mediated independently, and possibly at earlier stages of processing than dichoptic adaptation [63].

A further methodological consideration relates to our adaptor stimuli. Each block of adaptation involved the subject perceiving four grating patterns arranged in a 2×2 grid around fixation: one pair of gratings at one orientation; another pair othogonally oriented relative to the first. Although the relative local orientation of adaptor and test patterns was identical across testing blocks (at a given angular difference), the location of each of the adapting patterns was randomised prior to each testing block. An implication of this is that the global adaptor configuration varied from block-to-block. Although the present study has been concerned only with the effects local adaptation, future research might consider what role global adaptor configuration might play on subsequent local sensitivity.

### Functional implications

There are obvious benefits to be gained by the visual system preserving sensitivity in one eye whilst the other is differentially adapted (due to monocular half occlusions, for example). In fact, our results suggest that it may be possible to selectively preserve (or even improve) the sensitivity of a given eye by temporarily adapting it to zero contrast, whilst maintaining normal high-contrast viewing in the other eye. In a related vein, a recent study has show than short-term monocular deprivation can significantly improve detection thresholds in the deprived eye [64].

It has been suggested that the disproportionate reduction in low-spatial frequency sensitivity produced by cross-orientation masking reflects a divisive contrast normalisation process. The idea is that because natural scenes have a strong low-pass (1/f) spatial frequency bias a divisive contrast normalisation would serve to equalise visual responses (i.e. a whitening process), thereby reducing image redundancy and enhancing coding efficiency [8], [28], [65]–[66]. Our results suggest that an analogous (though purely monoptic) low spatial-frequency-specific sensitivity loss also occurs across time as a consequence of contrast adaptation.

Analogous to spatial frequency ‘whitening’, there may be a similar equalisation process for orientation, as supra-threshold orientation responses to natural stimuli have a broad but non-uniform orientation spectrum. In natural scenes, the contrast of horizontal and vertical components is greater than obliques because of gravity-driven pressures for structural stability [67]. [68] reported that orientation masking is greatest on cardinal axes, which produces an inverse-oblique effect that serves to normalise contrast responses across orientations. Future research is required to determine whether and to what extent adaptation may also whiten the orientation spectrum.

## Conclusions

We tested the orientation specificity of adaptation-induced threshold elevation at two spatial frequencies under monoptic and dichoptic conditions. The results may be summarised as a combination of isotropic and orientation-dependent effects. Significant and strong isotropic adaptation effects were observed under monoptic conditions and at the lowest spatial frequency tested. This set of contingencies suggests that isotropic adaptation is mediated by magnocellular-like mechanisms, possibly at a pre-cortical level of processing. By contrast, strongly orientation-dependent adaptation was found at both spatial frequencies tested under both monoptic and dichoptic conditions. Interestingly, the addition of the isotropic parameter to our von Mises models had the effect of stabilising orientation bandwidths as a function of spatial frequency, whilst broadening them under dichoptic conditions.

## Supporting Information

Appendix S1 **Control experiment in which orientation bandwidths are equated across stimulus spatial frequency.**
(DOCX)

Figure S1 **Mean monoptic threshold elevation estimates as a function of angular difference between adaptor and test stimuli with spatial frequencies of 0.25 and 2 c.p.d. (top and bottom respectively).** Data points are fitted with a standard von Mises model (Equation 1; dashed lines) and a von Mises model with an additive isotropic amplitude component (Equation 2; solid lines). Chi-square estimates of each fit are included for individual subjects and averaged data. Differences in chi-square greater than 3.84 are deemed statistically significant (*p*<.05, 1 degree of freedom).
(TIF)
