# Supplementary material for: Orientation-Specificity of Adaptation: Isotropic Adaptation Is Purely Monocular
Source: PLoS One. 2012 Nov 7;7(11):e47425. doi: 10.1371/journal.pone.0047425 (PMC3492394; doi:10.1371/journal.pone.0047425)
Supplement: Appendix S1 — Control experiment in which orientation bandwidths are equated across stimulus spatial frequency. (DOCX) [file pone.0047425.s001.docx]

Supporting Information

***Appendix 1***

A potential confound in this experiment arises from the fact that the visual angle of the stimulus envelopes was constant across spatial frequency. Consequently, there were four times fewer sinusoidal modulation cycles in the 0.25 c.p.d. compared with the 2 c.p.d. condition. To control for this variance (and any concomitant variation in stimulus orientation bandwidth) we conducted an additional experiment in which the number of cycles was maintained across spatial frequency.

**Method**

Two subjects with normal vision participated in this experiment. One was author JC and the other was naive to the purposes of the experiment. The experiment was identical in most respects to Experiment 1 except for the following details. All stimuli were viewed under purely monocular conditions without the aid of the Wheatstone stereoscope. Monocularity was achieved by placing an eye patch over the non-dominant eye. The diameter of the circular stimulus envelopes increased to 8° of visual angle for the 0.25 c.p.d. condition, and decreased to 2° of visual angle for the 2 c.p.d. condition. A single set of four adapting stimuli were presented in each of four 8° x 8° squares surrounding the central fixation point. From the observers’ perspective this was perceptually similar to Experiment 1 in so far as the subject perceived four adapting stimuli. As in Experiment 1, subjects first reported the location of the vertical target followed by the location of the horizontal target.

**Results**

As can be seen in Figure S1, Equation 2 produced a significantly better fit than Equation 1 at the lowest spatial frequency tested. Conversely, no significant differences were observed in the fits derived from Equations 1 and 2 at the higher spatial frequency.

**Conclusion**

Together, these results indicate that isotropic adaptation is evident under monocular conditions at the lowest spatial frequency tested and that there is no evidence for isotropic adaptation at the higher spatial frequency. Moreover, these results fail to support the hypothesis that the differences in isotropic adaptation observed as a function of spatial frequency in Experiment 1 were a consequence of differences in stimulus orientation bandwidth.
