# Supplementary figures and images for: Orientation-Specificity of Adaptation: Isotropic Adaptation Is Purely Monocular
Source: PLoS One. 2012 Nov 7;7(11):e47425. doi: 10.1371/journal.pone.0047425 (PMC3492394; doi:10.1371/journal.pone.0047425)

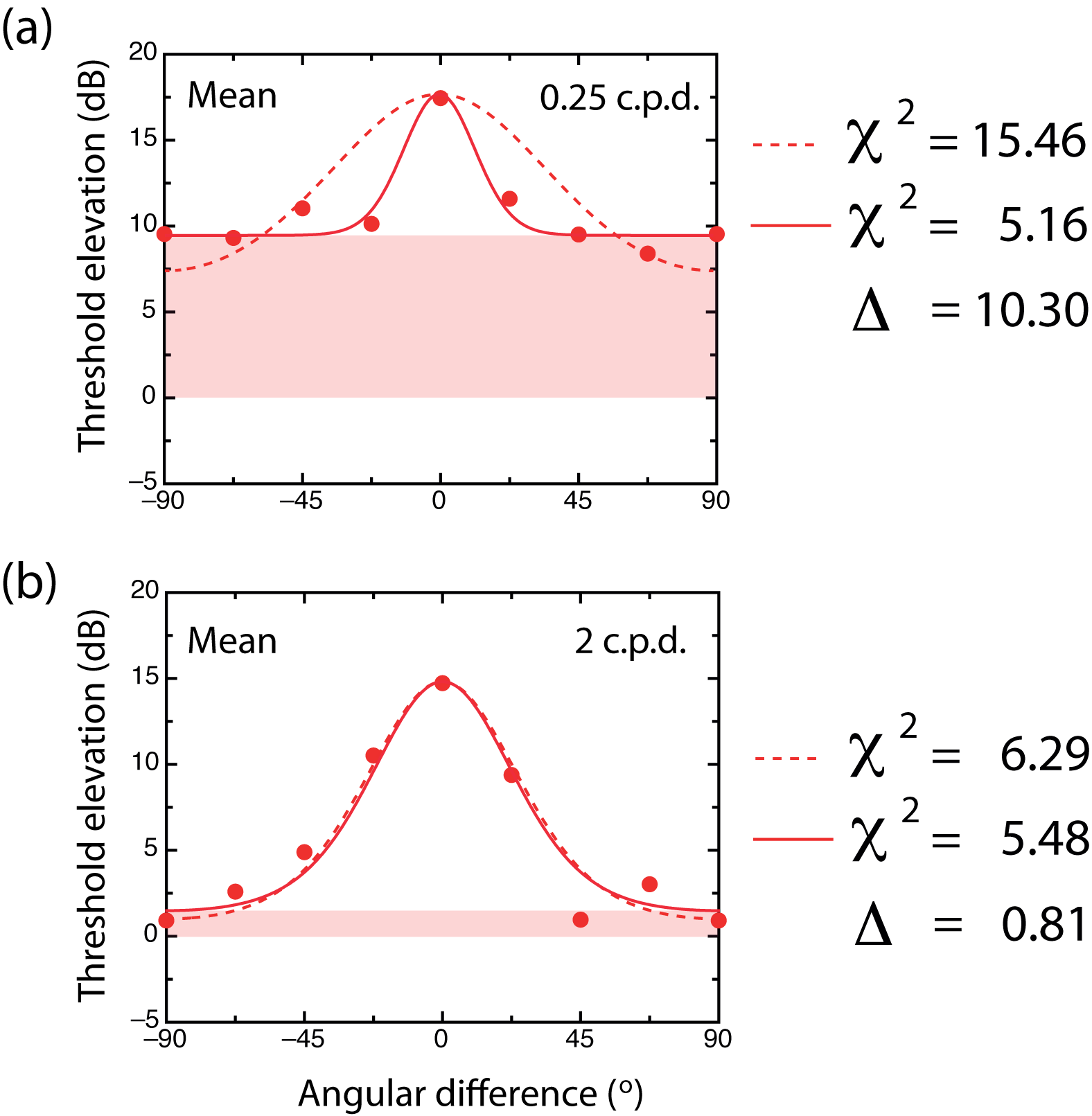

Supplement: Figure S1 — Mean monoptic threshold elevation estimates as a function of angular difference between adaptor and test stimuli with spatial frequencies of 0.25 and 2 c.p.d. (top and bottom respectively). Data points are fitted with a standard von Mises model (Equation 1; dashed lines) and a von Mises model with an additive isotropic amplitude component (Equation 2; solid lines). Chi-square estimates of each fit are included for individual subjects and averaged data. Differences in chi-square greater than 3.84 are deemed statistically significant (p<.05, 1 degree of freedom). (TIF) [file pone.0047425.s002.tif]
